# Whole‐exome sequencing facilitates the differential diagnosis of Ehlers–Danlos syndrome (EDS)

**DOI:** 10.1002/mgg3.1885

**Published:** 2022-02-04

**Authors:** Fang Yang, Rong‐Juan Yang, Qian Li, Jing Zhang, Yan‐Xin Meng, Xiao‐Jun Liu, Yong‐Feng Yao

**Affiliations:** ^1^ Department of Dermatology Shenzhen People's Hospital (The Second Clinical Medical College, Jinan University; The First Affiliated Hospital, Southern University of Science and Technology) Shenzhen China; ^2^ Candidate Branch of National Clinical Research Center for Skin Diseases Shenzhen China; ^3^ Department of Obstetrics Shijiazhuang Obstetrics and Gynecology Hospital Shijiazhuang China; ^4^ National Research Institute for Family Planning Beijing China; ^5^ Prenatal Diagnosis Center Shijiazhuang Obstetrics and Gynecology Hospital Shijiazhuang China; ^6^ Department of Intensive Care Unit Shenzhen People's Hospital (The Second Clinical Medical College, Jinan University; The First Affiliated Hospital, Southern University of Science and Technology) Shenzhen China

**Keywords:** *COL1A1*, *COL3A1*, *COL5A1*, Ehlers–Danlos syndrome, whole‐exome sequencing

## Abstract

Ehlers–Danlos syndromes (EDSs) are a group of rare monogenic conditions with strong heterogeneity and can be caused by 20 genes associating with the essence of the extracellular matrix (ECM). This study enrolled three cases with various subtypes of EDS. Clinical evaluation and genetic testing with whole‐exome sequencing (WES) were performed. The clinical manifestations of all three patients were thoroughly monitored; and three de novo diagnostic variants, namely *COL5A1*: NM_001278074.1: c.4609‐2A>C, *COL3A1*: NM_000090.3: c.3554G>T(p.Gly1185Val), and *COL1A1*: NM_000088.3: c.545G>T(p.Gly182Val) were identified from them, respectively. The findings in this study expanded the mutation spectrum of EDS and strengthened the efficiency of WES in the differential diagnosis on disorders with overlapping phenotypes and various pathogenesis.

## Graphical abstract

This study enrolled three cases with various subtypes of EDS. The clinical manifestations of all three patients were thoroughly monitored, and three de novo diagnostic variants in COL1A1, COL3A1, and COL5A1 genes were identified from them, respectively.

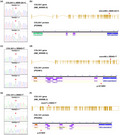

## 1 INTRODUCTION

The Ehlers–Danlos syndrome (EDS), first described by two dermatologists, Edvard Ehlers and Henri‐Alexandre Danlos, are a heterogeneous group of rare monogenic disorders mainly characterized by connective tissue friability, joint hypermobility, and skin and vascular fragility (Malfait et al., 2020). Patients with EDS commonly exhibit soft and hyperextensible skin, abnormal wound healing, and easy bruising. Complications of certain EDS subtypes, such as arterial aneurysm and dissection, can be severely life‐threatening (Malfait, 2018). There is still no specific medical or genetic therapeutic measure available for EDS, so integrated management and surveillance should be taken throughout the patients' lifetime.

The estimated prevalence of EDS is ~1 in 5,000 with no predisposition among ethnicities (Steinmann et al., 2002). So far, 14 various subtypes of EDS have been described, among which the genetic etiology of 13 was clarified, associating with 20 causative genes confirming to an autosomal dominant or recessive inheritance pattern (Malfait et al., 2017; Malfait et al., 2020). These genes include *COL1A1*, *COL1A2*, *COL3A1*, *COL5A1*, *COL5A2*, *ADAMTS2*, *PLOD1*, *FKBP14*, *TNXB*, *COL12A1*, *CHST14*, *DSE*, *B4GALT7*, *B3GALT6*, *SLC39A13*, *ZNF469*, *PRDM5*, *C1R*, *C1S*, and *AEBP1*, which all contribute to the essence of the extracellular matrix (ECM) by encoding or modifying fibrillar collagens types I, III, or V, or participating in the biosynthesis of the glycosaminoglycan (GAG) chains of proteoglycans (Malfait et al., 2020). The phenotypic overlap and genetic heterogeneity between various EDS subtypes pose a challenge in the clinical differential diagnosis of EDS, which is gradually solved in the wake of the rapid development of next‐generation sequencing (Joseph et al., 2018).

Classic EDS (cEDS, MIM #130000, and #130010), the most prevalent subtype, is mainly caused by pathogenic variants in *COL5A1* (MIM *120215) and *COL5A2* (MIM *120190) genes (over 90% cases), and also rarely by specific variations (certain “Arg to Cys” residue substitutions) in *COL1A1* (MIM *120150) (Malfait et al., 2007). Vascular EDS (vEDS, MIM #130050), caused by mutations in the *COL3A1* (MIM *120180) gene, is relatively the most severe subtype which could be lethal owing to vascular dissection or rupture, gastrointestinal perforation, or organ rupture (Byers, 1999). Another subtype, the arthrochalasia EDS (aEDS, MIM #130060, and #617821), is caused by mutations in *COL1A1* or *COL1A2* (MIM *120160) genes which would impact the proper N‐terminal cleavage of the peptides they encode and is distinguished from other types of EDS by the frequency of congenital hip dislocation and extreme joint laxity with recurrent joint subluxations and minimal skin involvement (Steinmann et al., 2002). Various subtypes of EDS, including the above three ones, together with other similar diseases may have similar phenotypic characteristics, so the differential diagnosis at molecular level is essential for their subsequent management.

In this study, we recruited three cases with patients exhibiting typical manifestations of EDS, and submitted them to genetic analysis with whole‐exome sequencing (WES). The findings in our study highlighted the capability of WES in achieving a definite diagnosis to various subtypes of EDS with overlapping symptoms.

## 2 MATERIAL AND METHODS

This study was approved by the Ethics Committee of Shijiazhuang Obstetrics and Gynecology Hospital (approval No.20210068), and written informed consent was obtained from all participants.

### 2.1 Subjects

Three unrelated cases, each with one patient exhibiting suspected EDS symptoms, were recruited between January/2018 and December/2020 at the department of dermatology, Shenzhen People's Hospital. These families were all Chinese Han ethnicity. A comprehensive physical examination was then conducted on the three patients.

### 2.2 Genomic DNA extraction

Three milliliters of peripheral blood was collected from the patients and their parents by means of BD Vacutainer™ tubes (BD Biosciences). Genomic DNA was extracted using the QIAamp DNA Blood Mini‐Kit (Qiagen Sciences), and the DNA quality was validated by 1% agarose gels and Qubit® DNA Assay Kit in Qubit® 2.0 Flurometer (Life Technologies).

### 2.3 Whole‐exome sequencing

Briefly, the enrichment of the exonic region sequences was conducted by the Sure Select Human Exon Sequence Capture Kit (Agilent). The sequencing libraries were quantified using the Illumina DNA Standards and Primer Premix Kit (Kapa Biosystems), and were massively parallel‐sequenced using the Illumina Novaseq6000 platform. After sequencing and filtering out the low‐quality readings, the high‐quality reads (with general quality level Q30 reads >89%) were compared to the human genome reference sequence [hg19]. The GATK software was used to identify suspected pathogenic variants (https://software.broadinstitute.org/gatk). The variations were identified by sequence alignment with the NCBI Reference Sequence (NG 011537.1) using Chromas v2.33. The pathogenicity of the identified variants was then assessed according to the common guidelines issued by the American Association of Medical Genetics and Genomics (ACMG) (Richards et al., 2015) referring to multiple databases (1000g2015aug_eas, https://www.internationalgenome.org/; ExAC_EAS, http://exac.broadinstitute.org; gnomAD_exome_EAS, http://gnomad.broadinstitute.org/); HGMD®: Human Gene Mutation Database (Professional Version 2019.4) with the Enliven® Variants Annotation Interpretation (Berry Genomics) system.

The suspected diagnostic variant was validated by Sanger sequencing using ABI 3730 Automated Sequencer (Applied Biosystems) according to the manufacturer's protocol.

### 2.4 Analysis of missense variants

The evolutionary conservatism of amino acid (AA) affected by specific missense variant was analyzed using MEGA7 (http://www.megasoftware.net) with default parameters.

## 3 RESULTS

### 3.1 Clinical manifestations

**FIGURE 1:**
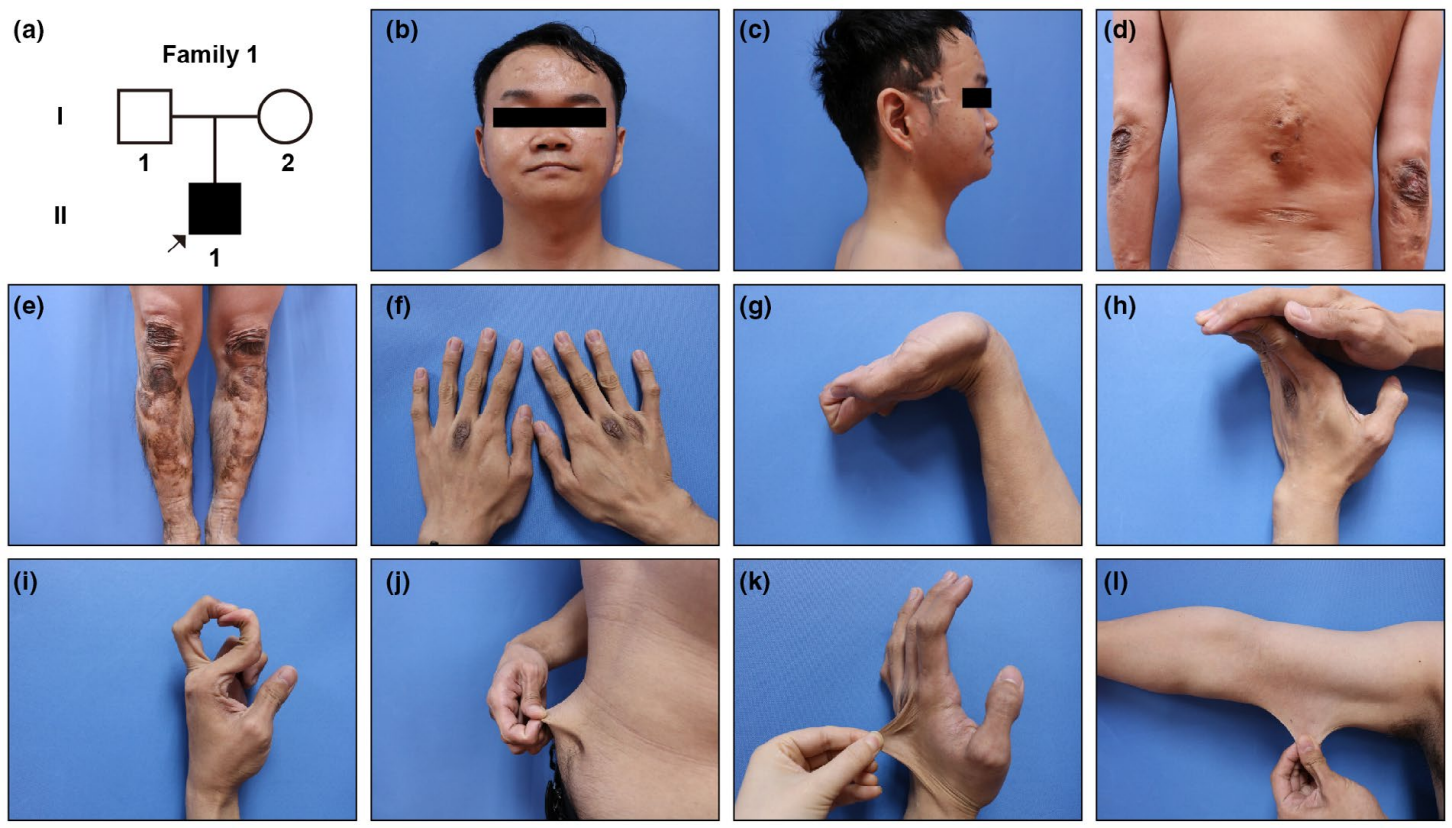
The pedigree diagram of Family 1 and manifestations of patient 1. (a) Pedigree diagram of case 1 (b–f) atrophic scars on forehead, temples, back, and shins, specific cicatrices after stretching of scars on the over‐pressure parts such as elbows, knees, and knuckles (g–i) hypermobile joints of wrists and fingers (j–l) hyperextensible skin

Case 1 The pedigree diagram of case 1 is depicted in Figure 1a. The male patient was 30 years old when he referred to our outpatient. His skin was fragile, so the areas prone to trauma of him (forehead, temples, back, and shins) were full of multiple atrophic scars (Figure 1b–e), and his over‐pressure parts (elbows, knees, and knuckles) manifested specific cicatrices after stretching of scars (Figure 1d–f). His joints of wrists and fingers were hypermobile (Figure 1g–i). His general skin was hyperextensible (Figure 1j–l). Based on these typical clinical indications, he was suspected to be with cEDS, and WES was therefore suggested.

**FIGURE 2:**
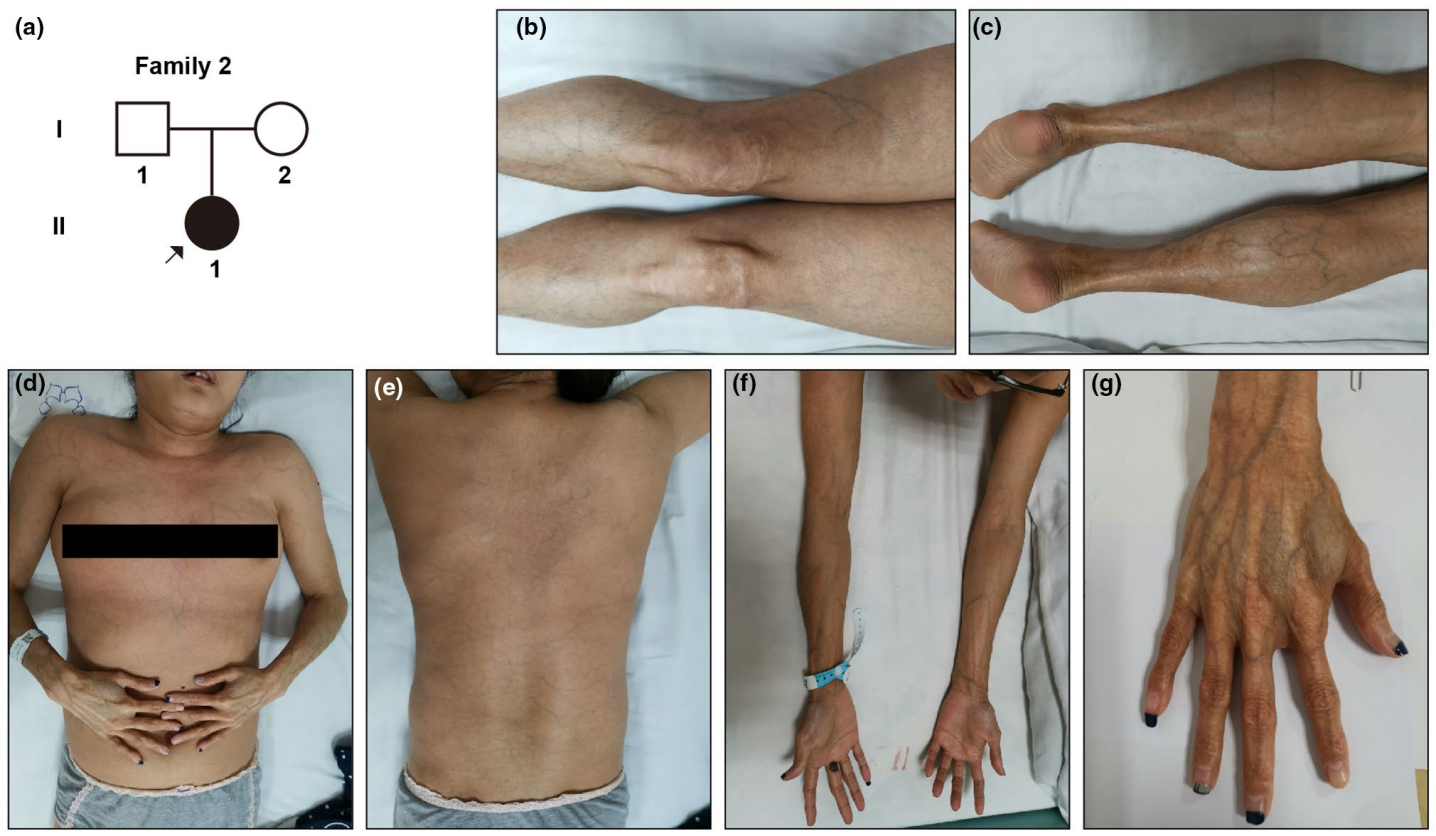
The pedigree diagram of Family 2 and manifestations of patient 2. (a) Pedigree diagram of case 2 (b–g) slender extremities, thin and translucent skin, talipesequinovarus, a visible venous pattern over limbs, chest, abdomen and extremities

Case 2 The pedigree diagram is depicted in Figure 2a. A 24‐year‐old female patient was admitted to our department with intermittent dizziness, increased blood pressure (BP:167/118 mmHg), and unexplained hypertension. Clinical and laboratory evaluation indicated that she suffered from insulin resistance, hypercholesterolemia, right renal infarction with perinephritis, superior mesenteric dissecting aneurysm, lung infection, and severe hepatic adipose infiltration. She showed slender extremities, thin and translucent skin, talipesequinovarus, a readily visible venous pattern over limbs, chest, and abdomen (Figure 2b–g). Two months after, she suddenly suffered from repeated dizziness and abdominal distension without inducement, accompanied by fatigue. As it progresses, the patient developed spontaneous celiac hemorrhage, small intestine necrosis, coagulation dysfunction, and splenic infarction. After laparoscopy, partial resection of the patient's small intestine was performed. Yet, the patient died from celiac hemorrhage in 24 hr. The patient's phenotype matched the vEDS, and we took her blood sample to conduct WES.

**FIGURE 3:**
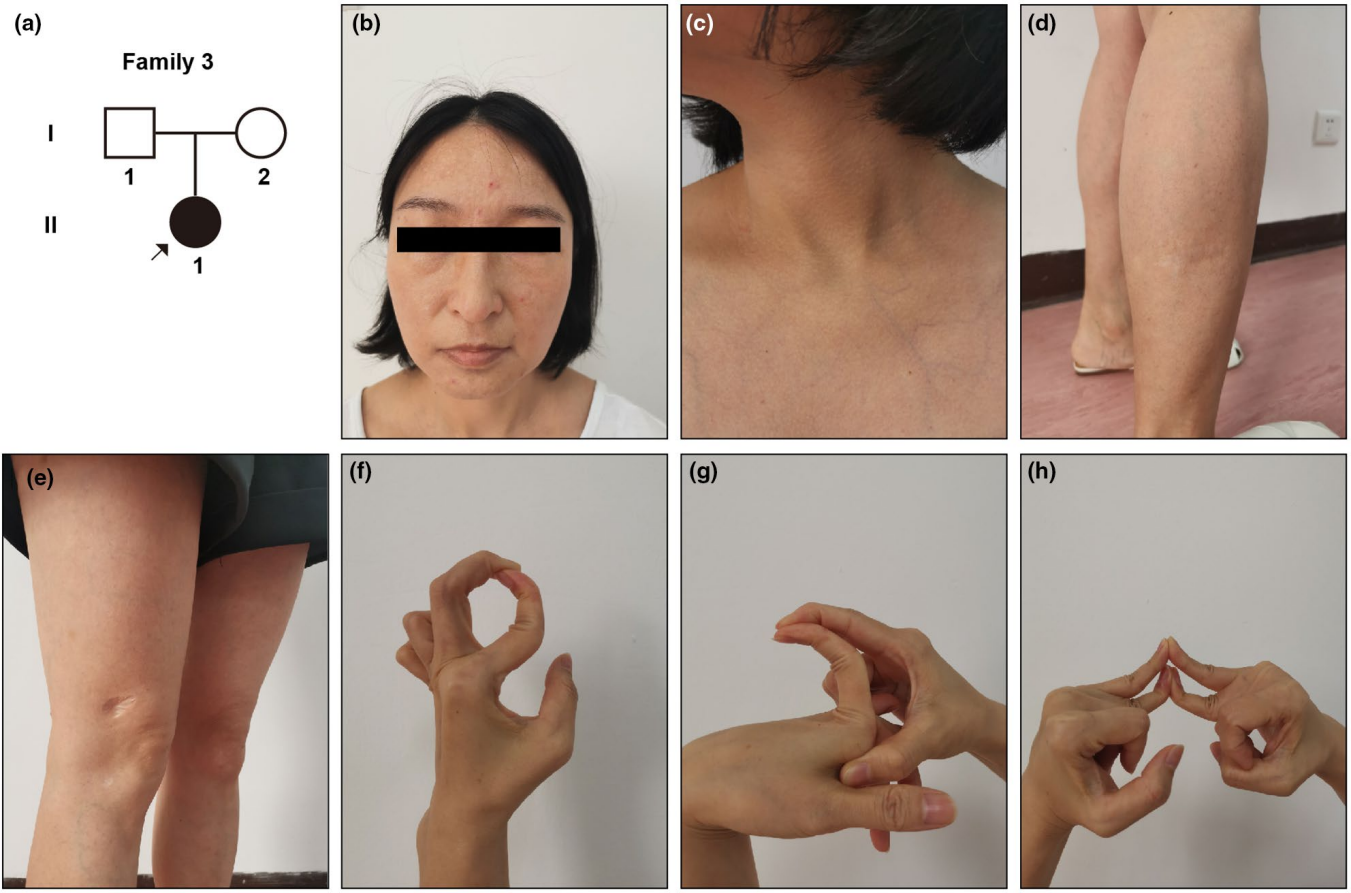
The pedigree diagram of Family 3 and manifestations of patient 3. (a) Pedigree diagram of case 3 (b–e) atrophic scars in her forehead, shin, and leg (f–h) hypermobile finger joints

Case 3 The pedigree diagram is depicted in Figure 3a. The female patient was 33 when she referred to our department. There were some spots of atrophic scars on her forehead, shin, and leg (Figure 3b, d and e), yet it was less severe than those in Case 1 patient. The skin in her prothorax showed mild translucency (Figure 3c). Her finger joints were also hypermobile (Figure 3f–h), but her skin was with less extensibility than typical cEDS. According to the patient, she had no history of joint dislocation or cryptogenic bone fractures.

### 3.2 Genetic variations

**FIGURE 4:**
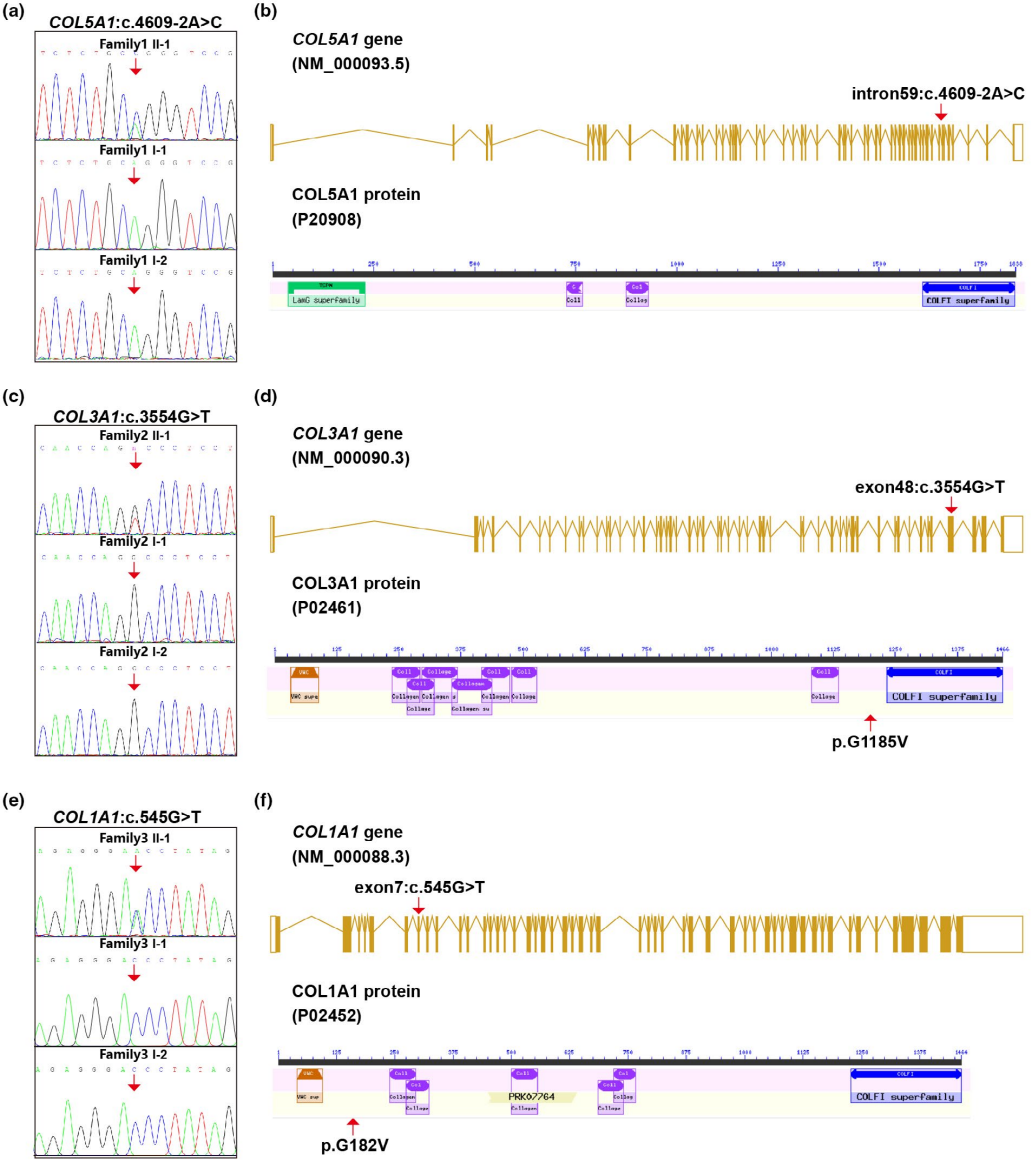
Genetic variants detected in the three cases. (a) A de novo splicing site variant, *COL5A1*: c.4609‐2A>C in Patient 1 (Family 1 II‐1). (c) A de novo missense variant, *COL3A1*: c.3554G>T in Patient 2 (Family 2 II‐1). (e) A de novo missense variant, *COL1A1*: c.545G>T in Patient 3 (Family 3 II‐1). (b, d, and e) showing the location of each variant in respective gene and peptide diagrammatic sketches

According to WES results, all three patients were positive with heterozygous variants (detailed data in Table 1). Patient 1 (II‐1 in Family 1) carried a splicing site variant, namely *COL5A1*: NM_001278074 0.1: c.4609‐2A>C (Figure 4a); Patient 2 (II‐1 in Family 2) carried a missense variant, *COL3A1*: NM_000090.3: c.3554G>T(p.Gly1185Val) (Figure 4c); and Patient 3 (II‐1 in Family 3) carried a missense variant, *COL1A1*: NM_000088.3: c.545G>T(p.Gly182Val) (Figure 4e). Based on the following familial validation using Sanger sequencing, it was demonstrated that all these variants were de novo (Figure 4a, c, and e). The location of each variant was illuminated in the gene and peptide diagrammatic sketches (Figure 4b, d and f).

**TABLE 1:** Variation characterization in this study

Patient no.	Gene^①^	Exon/intron	DNA variant	Protein variant	Variation frequencies In 3 databases^②^	Revel_Score^③^	HGMD^④^	PMID^⑤^	Level (Evidence)^⑥^
Family1II‐1	*COL5A1*	intron59	c.4609‐2A>C		0; 0; 0	—	—	—	Likely pathogenic (pvs1 + pm2)
Family2II‐1	*COL3A1*	exon48	c.3554G>T	p.G1185V	0; 0; 0	0.999	DM	9,036,918	Likely pathogenic (pp2 + pm2 + pm5 + pp3)
Family3II‐1	*COL1A1*	exon7	c.545G>T	p.G182V	0; 0.00000834; 0.00000798	0.974	—	—	VUS (pp2 + pm2 + pp3)

*Note*: ①Transcript ID: *COL5A1* (NM_000093.5);*COL3A1* (NM_000090.3);*COL1A1* (NM_000088.3); ②1000 genomes (https://www.internationalgenome.org/); ExAC (http://exac.broadinstitute.org); gnomAD_exomes (http://gnomad.broadinstitute.org/); ③An ensemble method for predicting the pathogenicity of missense variants on the basis of individual tools: MutPred, FATHMM, VEST, PolyPhen, SIFT, PROVEAN, MutationAssessor, MutationTaster, LRT, GERP, SiPhy, phyloP, and phastCons (https://doi.org/10.1016/j.ajhg.2016.08.016); ④HGMD®: Human Gene Mutation Database (Professional Version 2019.4); ⑤PMID: PubMed ID (https://pubmed.ncbi.nlm.nih.gov/); ⑥ACMG: The American College of Medical Genetics and Genomics; P: pathogenic; LP: likely pathogenic; VUS: variants of unknown significance; LB: likely benign.

### 3.3 Conservatism analysis of missense variants

**FIGURE 5:**
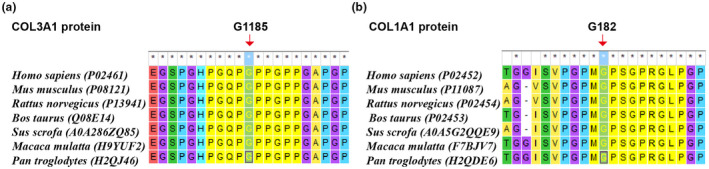
The evolutionary conservatism of the two affected amino acids, (a) COL3A1: Gly1185 and (b) COL1A1: Gly182 among species

As described above, two missense variants were detected in this study. The evolutionary conservatism of AAs affected by them were analyzed. Resultantly, it was indicated the AAs, namely COL3A1: Gly1185 and COL1A1: Gly182, maintained conserved across species (Figure 5).

## 4 DISCUSSION

EDSs and other disorders of joint hypermobility have been described and studied for over 100 years (Chernogubow, 1892; Ehlers, 1901). For some common EDS subtypes, in addition to routine clinical diagnosis and management, genotype–phenotype correlation began to emerge owing to an increasing number of studies and identified variants (Malfait et al., 2020; Paladin et al., 2015; Rohrbach et al., 2011; Weerakkody et al., 2016). Besides, the phenotypic expressivity and environmental influence are being better elucidated since more studies involving in vitro models, transcriptome, and proteome were carried out (Chiarelli et al., 2018; Chiarelli et al., 2019).

In this study, we presented three cases with various EDS situations. Indicated by the clinical and genetic findings, Patient 1 was a typical cEDS sufferer. Up to date, more than 200 distinct pathogenic variants in *COL5A1* and *COL5A2* have been identified, accounting for over 90% cEDS cases (Ma et al., 2021). A novel variant at the splicing site, *COL5A1*: c.4609‐2A>C, was detected in Patient 1. According to the general standard issued by ACMG (American Committee of Medical Genetics and Genomics) (Richards et al., 2015), it was inferred to be “Pathogenic” with the evidence levels of “PVS1 + PM2 + PP4”. However, how it affected the *COL5A1* mRNA splicing is still to be investigated, and may be of particular importance for the contribution to genotype–phenotype correlation of cEDS.

vEDS is the most serious EDS subtype, with an incidence of about 1/900,000, and a median survival age at 54 years (Byers, 1999). So far, more than 480 pathogenic variants in *COL3A1* gene have been indexed to cause vEDS (http://www.hgmd.cf.ac.uk), among which, patient with exon‐skip variants would have the minimum age of survival; the substitution of glycine in “Gly‐Xaa‐Yaa” triplet repeats, particularly by glutamic acid or valine, could cause worse phenotype than substitution by smaller AAs (Malfait, 2018; Mizuno et al., 2013; Pepin et al., 2014). In this study, Patient 2 exhibited visible skin translucency, which is a characteristic feature of vEDS (Brady et al., 2017); and a reported missense variant, *COL3A1*: c.3554G>T(p.Gly1185Val), located in the N‐terminal “Gly‐Xaa‐Yaa” triplet repeat domain of COL1A1, was detected in her (Pepin et al., 2014). According to the ACMG criteria, it was determined as “likely pathogenic” (Table 1). In this patient, some uncommon symptoms like unexplained hypertension, insulin resistance, and hyperlipidemia emerged simultaneously. Gerogiannis et al. recently described another senior patient with similar metabolic manifestations (Gerogiannis et al., 2020). Further study is needed to clarify the functional impact of defective type III collagen resulting in these indications. Additionally, due to the lethal nature of vEDS, there are also some promising therapeutic studies, such as to use of MMP inhibitors (Tae et al., 2012). But there is still a long way to go till their clinical utilization.

The *COL1A1* gene exhibits strong pleiotropy and contributes to the pathogenesis of a greater part of osteogenesis imperfecta (OI) and aEDS cases, and also a few cases of cEDS or vEDS (Malfait et al., 2020; Marini et al., 2017). Moreover, mutations in the amino end of type I collagen could even result in OI/EDS combined syndrome (Cabral et al., 2005). This is probably because the type I collagen is the major protein component of the ECM in many tissues such as the bone, dermis, blood vessel walls, and tendon, and co‐assembles with type V or type III during the collagen fibril formation process (Malfait et al., 2020). In our study, we detected a novel missense variant, *COL1A1*: c.545G>T(p.Gly182Val) in Patient 3. Although according to the ACMG criteria (pp2 + pm2 + pp3), we can only determine this variant as VUS (variation of uncertain significance) by now, its pathogenicity should be further determined by functional study and/or tissue biopsy, since no other suspiciously causative variant was detected in the coding regions of any of the 20 EDS genes (data not shown). Patient 3 denied a history of congenital hip dislocation and multiple recurrent dislocation, so it did not meet the aEDS characteristics. Based on the patient's clinical indications, we cannot exclude her as a cEDS/vEDS overlapping subtype. So, attention should be continuously paid to her risk of vascular complications. It was reported that most cases of aEDS caused by *COL1A1* or *COL1A2* were due to the abnormal N‐terminal propeptide cleavage (Byers et al., 1997). Since the Gly182Val variant is located in the N‐terminal “Gly‐Xaa‐Yaa” triplet repeat domain, how it affects the structure and function of the collagen I peptide chain requires further investigation, which may reveal the specific mechanism of its pathogenesis.

Two missense variants in this study both have low incidence (Table 1). And the evolutionary conservatism of the two AAs affected by the missense indirectly supports their pathogenicity. Besides, the predictive analysis scores of them by Revel were both over 0.9 (Table 1, cutoff value is ≥0.7) supporting them to be deleterious. The main limitation of this study was the lack of functional experiments to explore the pathogenic mechanism of novel variants. Besides, it only reported individual cases, so the genotype–phenotype correlation could not be established appropriately.

In summary, we report three EDS cases with various genetic etiologies and different clinical manifestations. The findings in this study expanded the mutation spectrum of EDS, provided solid evidence for the counseling to the affected families, and might shed light on the pathogenesis of various collagenosis.

## CONFLICT OF INTEREST

The authors declare that there is no conflict of interest regarding the publication of this paper.

## AUTHORS’ CONTRIBUTIONS

FY designed this study and wrote this manuscript, and RjY reviewed and corrected it. QL analyzed experimental data and composed the figures and Tables. FY and YfY recruited the case and did the clinical examination. JZ, YxM, and XjL performed the genetic experimental and in silico studies.

## Data Availability

The underlying data supporting the results of this study can be required to the corresponding author based on reasonable demand.
